# Visible light focusing flat lenses based on hybrid dielectric-metal metasurface reflector-arrays

**DOI:** 10.1038/srep45044

**Published:** 2017-03-23

**Authors:** Qingbin Fan, Pengcheng Huo, Daopeng Wang, Yuzhang Liang, Feng Yan, Ting Xu

**Affiliations:** 1 National Laboratory of Solid State Microstructures, College of Engineering and Applied Sciences and Collaborative Innovation Center of Advanced Microstructures, Nanjing University, 22 Hankou Road, Nanjing 210093, China; 2 School of Electronic Science and Engineering, Nanjing University, 22 Hankou Road, Nanjing 210093, China

## Abstract

Conventional metasurface reflector-arrays based on metallic resonant nanoantenna to control the wavefront of light for focusing always suffer from strong ohmic loss at optical frequencies. Here, we overcome this challenge by constructing a non-resonant, hybrid dielectric-metal configuration consisting of TiO_2_ nanofins associated with an Ag reflector substrate that provides a broadband response and high polarization conversion efficiency in the visible range. A reflective flat lens based on this configuration shows an excellent focusing performance with the spot size close to the diffraction limit. Furthermore, by employing the superimposed phase distribution design to manipulate the wavefront of the reflected light, various functionalities, such as multifocal and achromatic focusing, are demonstrated for the flat lenses. Such a reflective flat lens will find various applications in visible light imaging and sensing systems.

Manipulating the flow of photons has become a vital research topic with the development of integrated optical circuit and optical communication systems in recent years. Metasurfaces, composed of phase shifters formed by the subwavelength nanostructures at a flat surface, have attracted a lot of attentions due to their gorgeous performances and ultrathin thickness compared to conventional bulk optical components^1^,^2^,^3^. Metasurfaces are able to arbitrarily control polarization, phase, and amplitude of incident light. Thanks to these advantages, various metasurface-based optical devices have been implemented including flat lens^4^,^5^,^6^,^7^,^8^,^9^, beam deflectors^10^,^11^, wave plates^12^,^13^, vortex generators^14^,^15^,^16^ and holograms^17^,^18^,^19^,^20^. In general, metasurfaces can be divided into two categories: plasmonic metallic metasurfaces^5^,^6^ and all-dielectric metasurfaces^7^,^8^. Recently, low-loss dielectric metasurfaces have been proposed to achieve a transmissive flat lens by using titanium dioxide (TiO_2_) in the visible spectrum^21^,^22^. As one of the most fundamental components in the field of optics, these metasurfaces-based planar focusing lens breaks the thickness limit of conventional lens. Focusing light by reflective lens is an alternative to the transmissive one. However, the reflective lens usually employs a resonant metal-dielectric-metal (MDM) architecture and they have limited efficiencies due to ohmic loss, typically lower than 30% for the visible and near-infrared light^5^,^23^. Although many efforts have been paid in designing reflective lens, there are still many aspects needing improvement, such as polarization conversion efficiency, focal spot size, operation bandwidth and chromatic aberrations, especially for the visible light^5^.

In this letter, we propose and numerically demonstrate that a non-resonant, hybrid dielectric-metal configuration consisting of dielectric nanofins associated with metallic mirror substrate can be used to build a metasurface element and work as a phase shifter for the desired phase profile. The hybrid structure exhibits a broadband (590~720 nm) optical response and high polarization conversion efficiency (higher than 80%). Based on this geometry, a reflective flat lens is designed in visible range, which is capable of focusing energy at arbitrary position above the metasurface though tuning phase profile formed by reflector-arrays. Furthermore, by designing a meta-molecule comprising two sets of different nanofins, the reflective flat lens is able to achieve various optical functionalities including multifocal and achromatic focusing.

## Results

**Figure 1:**
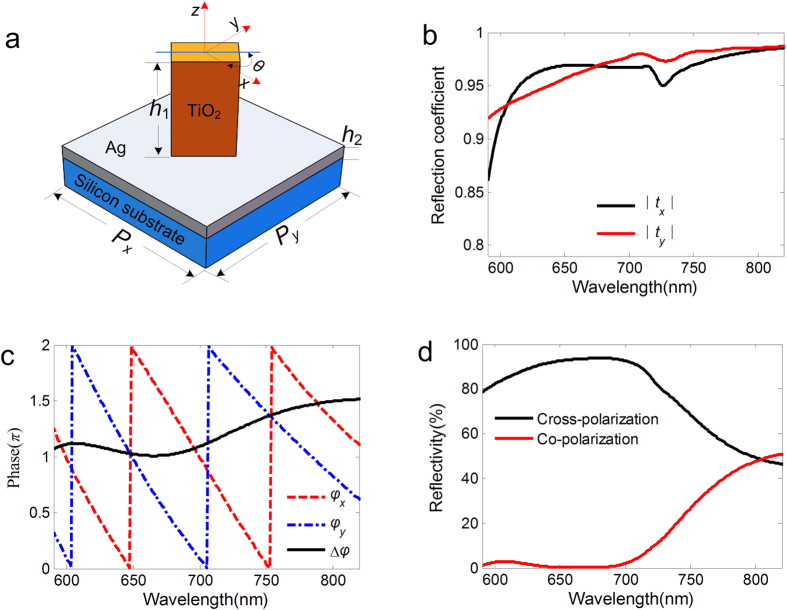
(**a**) Schematic of the unit-cell structure with *P*_*x*_ = *P*_*y*_ = 250 nm. The flat lens consists of TiO_2_ nanofins on an Ag mirror. The nanofin can rotate in the *x-y* plane with an angle *θ* to create a required phase according to the Pancharatnam-Berry phase. The nanofin have length *L* = 190 nm, width *W* = 90 nm and height *h*_1_ = 600 nm. The Ag film has thickness of *h*_2_ = 150 nm. (**b**) Calculated reflection coefficients for *x*- and *y*-polarized light and (**c**) their phase *φ*_*x*,_ *φ*_*y*_ and phase difference Δ*φ*. (**d**) Calculated reflectivity of the cross-polarization and co-polarization for the designed hybrid structure.

The fundamental unit cell of the designed metasurface is a hybrid dielectric-metal structure consisting of an amorphous TiO_2_ nanofin on a silver mirror substrate, as shown in Fig. 1(a). TiO_2_ is chosen here due to its sufficiently high refractive index, low surface roughness, especially low loss at visible frequencies^21^,^22^. Two principles are taken into account for determining the period of the unit cell. First, the period size should be smaller than the visible operation wavelength so that the naonfin structure can efficiently control the wavefront of light. Second, the period size should be realistic and can be fabricated with routine nanofabrication techniques, such as electron beam lithography^21^. It is well known that the condition of the inter-conversion of left/right circular polarization (LCP/RCP) is to generate a phase delay of π between the *y*-polarized component and the *x*-polarized component. Thus, to achieve high polarization conversion efficiency which is required for effective manipulation of reflected light, the phase difference between the reflection of *x* and *y*-polarized components of the nanofin should be equal to π. In addition, the reflection amplitudes of the both linear polarization states need maintain large and equal values. Figure 1(b and (c)) show the amplitude of reflection coefficients of the cross-polarization and their phase and phase difference, respectively. The phase difference between two reflection coefficients approaches π within wide spectral range 590–720 nm. At the same time, the configuration maintains a weak variation in the reflection amplitudes for both linear polarizations. The cross-polarization has the opposite chirality as the incident circularly polarization light with an additional phase, which can be controlled to achieve various functionalities. The co-polarization has the same chirality as the incident circularly polarization light without an additional phase, maintaining original direction of propagation. In our design, the polarization conversion efficiency from RCP/LCP to cross-polarization is over 80% within the investigated wavelength range, as shown in Fig. 1(d).

**Figure 2:**
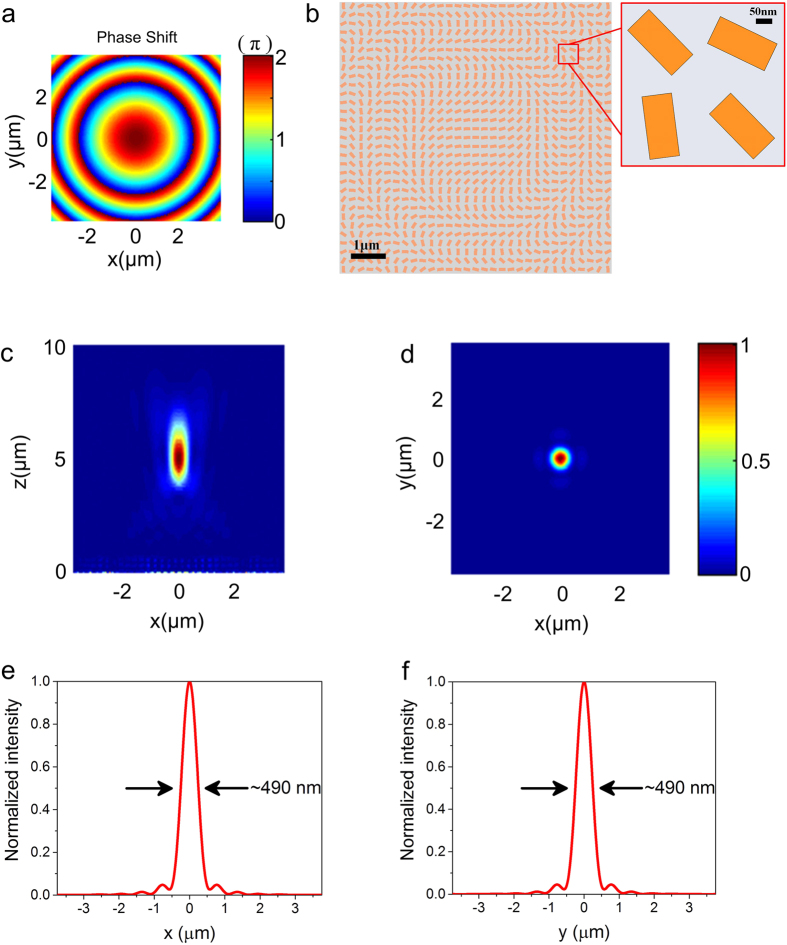
(**a**) The ideal phase shift distribution of the designed lens for the normal incident light. The phase profile of the lens is obtained by changing the orientation angle *θ* of the individual nanofin. (**b**) Structure of the flat lens designed at the wavelength of 650 nm with a focal length *f* = 5 μm. The inset shows a zoomed-in image of several local nanofins. (**c**) Simulated electric intensity distribution of the reflected beam in the *x-z* plane at *y* = 0 and (**d**) *x-y* plane at the focal point. The normal incident beam is RCP light. (**e,f**) The normalized intensity cross-sections at the focal spot center along the *x* and *y* axis. FWHMs of the focal spot along two directions are labelled on the plots.

A geometric phase, or called Pancharatnam-Berry phase, is picked up by spatially rotating the nanofin to generate a phase shift 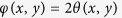, where *θ* is the orientation angle of the nanofin^24^. A major advantage of geometric metasurface is that it mainly depends on rotation angle and is insensitive to the nanofin size variations and operating wavelength. Using the unit cell configuration as a pixel cell to cover the whole phase space from 0 to 2π, one can achieve desired optical functionality by designing the metasurface array’s phase profile. To construct a reflective flat lens with a focal length *f* for the normal incident light, the phase shift φ imposed on each point of the reflector-arrays relies on the following phase profile:


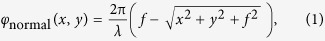


where (*x, y*) is the central coordinate of each unit cell and *λ* is the wavelength in free space. The ideal phase profile *φ*_normal_ is ploted as color-scale in Fig. 2(a) for a focal length of 5 μm working at the wavelength of 650 nm. The distribution of the phase should be discretized on the metasurface, corresponding to the center phase of each unit cell. Figure 2(b) shows the designed flat lens with 31 × 31 unit cells, where the inset is a zoomed image of several local nanofins. The flat lens provides a strong focusing capability with a numerical aperture (N.A.) of ~0.74. The normal incident RCP light propagates to the reflector-arrays, converts to LCP light and focuses light to the designed position. Figure 2(c) and (d) depict the finite-difference time-domain (FDTD) simulated intensity (normalized 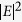) of the reflected beam in *x-z* and *x-y* plane, respectively, showing a perfect focusing performance with full-width at half-maximum (FWHM) of 490 nm (Fig. 2(e) and (f)) and the spot size is close to the diffraction limit.

**Figure 3:**
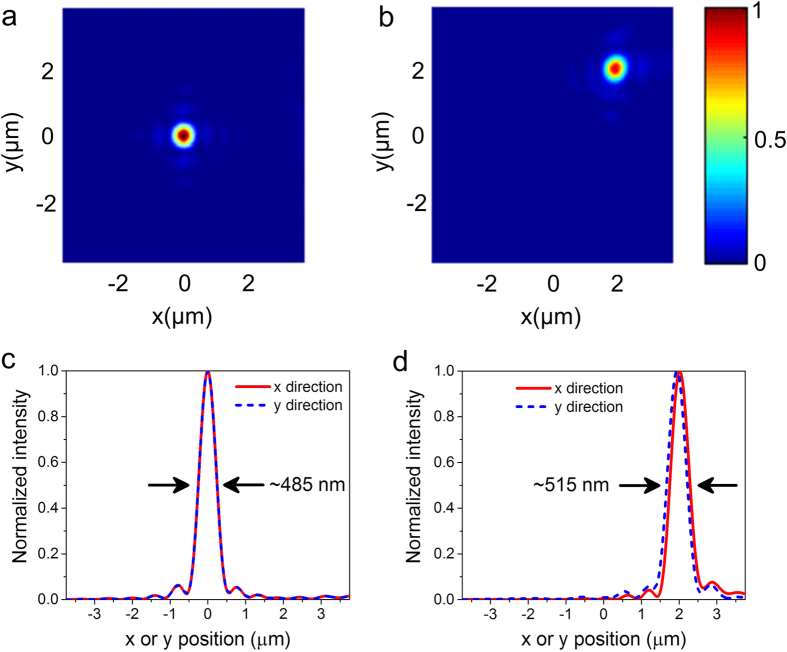
Simulated electric intensity distributions of the reflected beams in *x-y* plane at the focal point ( *f* = 5 μm) for (**a**) center position (*x* = 0, *y* = 0) and (**b**) arbitrary position (*x* = 2 μm, *y* = 2 μm). The oblique incident beam is RCP light with an incident angle of *α* = 45°. (**c,d**) The normalized intensity cross-sections at the focal spot center along the *x* and *y* axis for center position (**c**) and arbitrary position (**d**). FWHMs of the focal spots are labelled on the plots.

In order to show the control ability of the hybrid structure for the oblique incident light, we design two lens to focus oblique incident light at arbitrary positions in *x-y* plane. Assuming a circular polarization light illuminates the flat lens with the oblique incident angle *α*, then reflects and focuses at arbitrary position (*x*_0_, *y*_0_) in *x*-*y* plane, a total phase shift *φ* could be given as:


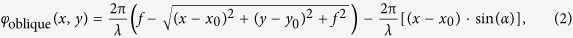


where the additional phase profile 
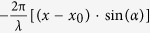
 compensates for oblique incident light arriving with a different optical length. At the operation wavelength of 650 nm, focal length of 5 μm and incident angle of *α* = 45°, two phase profiles *φ*_oblique_ with continuous phase distribution are calculated and they are discretized by nanostructures (see Supplementary Fig. S1 for phase distribution and corresponding structure of the flat lens). The phase profile is designed to focus light at central position (*x*_0_ = 0, *y*_0_ = 0, Fig. S1(a)) and at arbitrary position (here we choose *x*_0_ = 2 μm, *y*_0_ = 2 μm, Fig. S1(b)). The simulated focusing electric intensity of the corresponding devices are shown in Fig. 3(a) and (b), respectively. The simulation results show good consistence with the designed focus positions, and the focal spots have FWHM of 485 nm and 515 nm for two devices, as shown in Fig. 3(c) and (d). Besides high cross-polarization conversion efficiencies and good focusing performances, compared with previous studies, our reflective flat lens based on hybrid dielectric-metal geometry metasurface is easier to be realized in the visible range and able to achieve two-dimensional focusing as a spot, rather than one-dimensional focusing as a line^5^,^9^.

**Figure 4:**
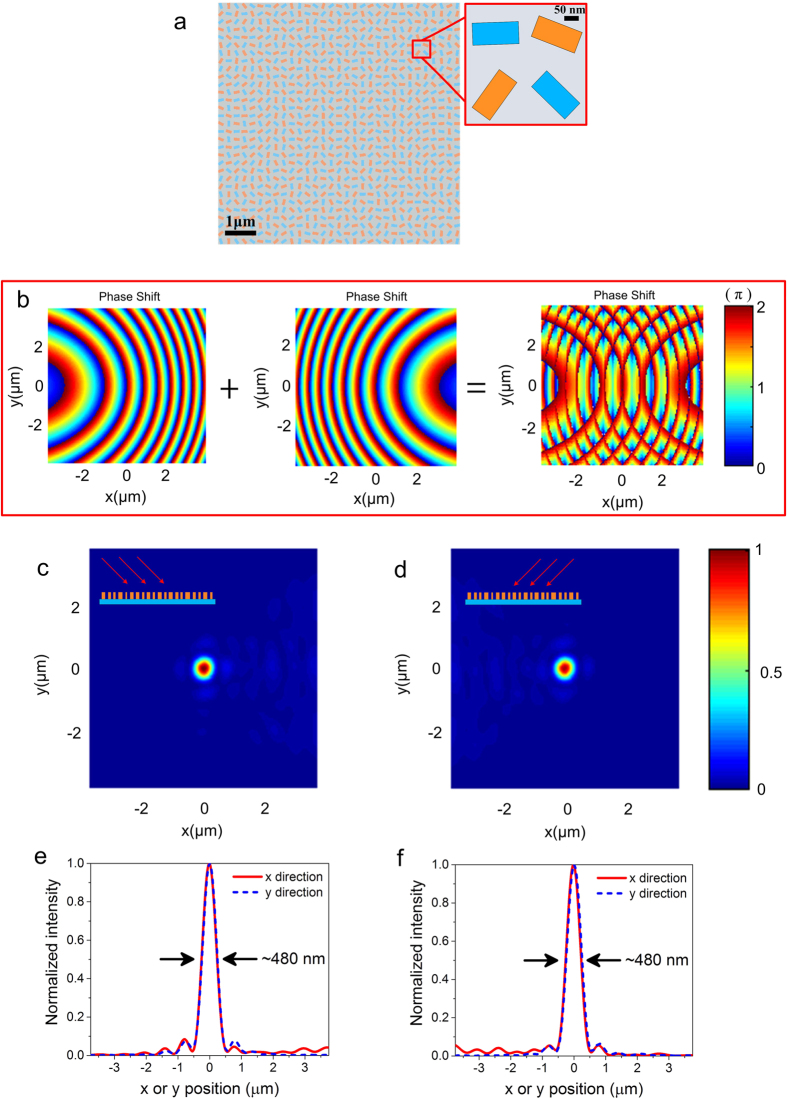
Illustration of the superimposed phase to produce a multifunction metasurface. (**a**) Schematic diagram of the composited flat lens. The inset shows a zoomed-in meta-molecule. Structural parameters of the nanofins are consistent with above. (**b**) Illustration of the formation principle of the superimposed phase. The desired phase distribution is formed by the superposition of two sub-phases, in which one is to compensate the phase of right oblique incident light with *α* = 45° and the other is to compensate the phase from opposite direction. (**c,d**) Simulated electric intensity distributions of the focal spot in *x-y* plane for *α* = 45° (**c**) and *α* = −45° (**d**). (**e,f**) Corresponding normalized intensity cross-sections at the focal spot center along the *x* and *y* axis for *α* = 45° (**e**) and *α* = −45° (**f**). FWHMs of the focal spots are labelled on the plots.

For a designed metasurface lens, it typically has the best response to a specific direction of incident light, while the other direction of the incident light will result in diverging. Here, a reflective flat lens able to focus incident light from different directions is shown in Fig. 4(a). It consists of two interlaced arrays of khaki and blue nanofins with the same size but different rotation angles, producing two types phase profile multiplexed. The inset in Fig. 4(a) shows a zoomed-in meta-molecule, containing two types of nanofins represented by different colors to control two different phase profiles. Figure 4(b) is an example to illustrate that the new phase distribution on the metasurface is superimposed by two different phase profiles, one of which is used to focus oblique light incident from left at *θ* = 45° and the other is used to focus oblique light incident from the opposite direction. It should be noted that this method can easily be extended to design composite flat lens to focus light from more directions, which can not be easily achieved by the traditional dielectric lens. Simulation results shown in Fig. 4(c)–(f) demonstrate a good focusing performance of the reflective flat lens at the origin position for two different incident directions and agrees well with our design.

## Discussion

**Figure 5:**
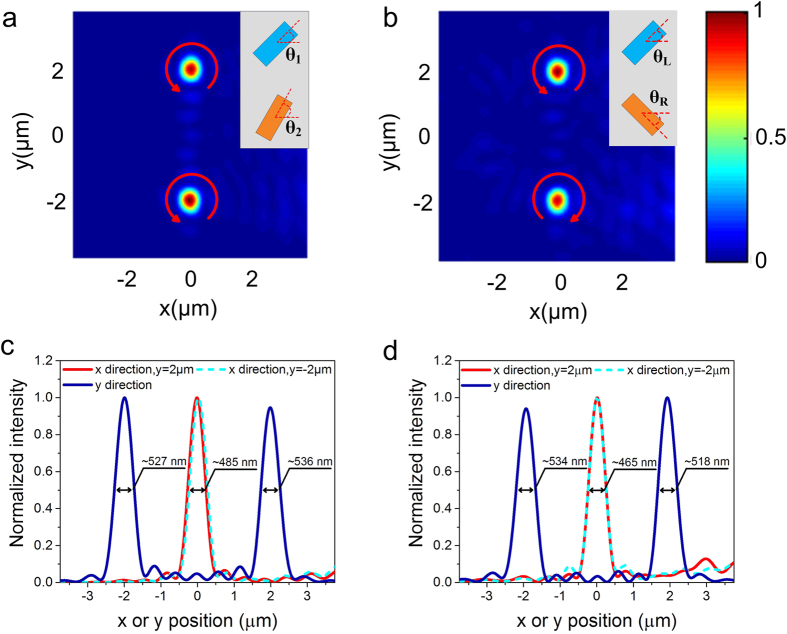
Simulated electric intensity distribution of the reflected beam (*f* = 5 μm). (**a**) Two focal points with the same chirality formed by meta-molecule achiral lens (Two kinds of nanofins showed in Fig. 4(a) with the same rotational directions). The oblique incident beam is RCP light. The distance between the two focal points is 4 μm. The red arrow represents helicity of focused light. (**b**) Two focal points with the opposite chirality formed by meta-molecule chiral lens (Two kinds of nanofins showed in Fig. 4(a) with opposite rotational directions). The oblique incident beam is linear polarized light. (**c**) and (**d**) Corresponding normalized intensity cross-sections at the flat lens’s focal spots along the *x* and *y* axis for the same chirality (**c**) and opposite chirality (**d**). FWHMs of the focal spots are labelled on the plots.

By employing the superimposed phase design idea, various functionalities can be achieved such as multifocal focusing with the same or opposite chirality^25^,^26^,^27^ and achromatic focusing^28^,^29^,^30^,^31^. For multifocal focusing with the same chirality, such as RCP light, according to the Eq. 2, the required phase profiles for the two designed focusing position 1 (*x* = 0 μm, *y* = 2 μm) and 2 (*x* = 0 μm, *y* = −2 μm) are achieved by simultaneously counterclockwise rotating the nanofins with different angles (see Supplementary Fig. S2(a) and (c) for the phase distribution and corresponding structure of the flat lens). As expected, the incident RCP light is then converted to LCP light by the flat lens and focuses at two positions (*x* = 0 μm, *y* = 2 μm and *y* = −2 μm) in *x-y* plane, as shown in Fig. 5(a) and (c).

On the other side, for multifocal focusing with opposite chirality, the required geometric phases are imparted by rotating the nanofins with opposite directions (see Supplementary Fig. S2(b) and (d) for the phase distribution and corresponding structure of the flat lens). For incident RCP (LCP) light, the nanofins are counterclockwise (clockwise) rotated with an angle *θ*_L_ (*θ*_R_) to compensate the required phases. Therefore, the focusing lens is composed of two individual lens 1 and 2 which are designed for the RCP and LCP incident light, respectively, similar to the schematic diagram shown in Fig. 4(a). In order to demonstrate the multifocal focusing with opposite chirality, here we use a light source with linear polarization along the *x*-axis which is equivalent to a superposition of both circular polarized light components. The incident linearly-polarized light propagates to the reflector-arrays, in which lens 1/lens 2 responses to RCP/LCP components, respectively, converting them to the LCP/RCP light and focusing at the designed positions (*x* = 0 μm, *y* = 2 μm and *y *= −2 μm). The multifocal focusing with opposite chirality is numerically demonstrated in Fig. 5(b)and (d). One thing to note here is that the energy of each focal point is only assigned less than a quarter of the incident light, because such superimposed phase enables each nanofin only to focus one type of chiral circular polarized light and diverge the opposite chiral light simultaneously.

**Figure 6:**
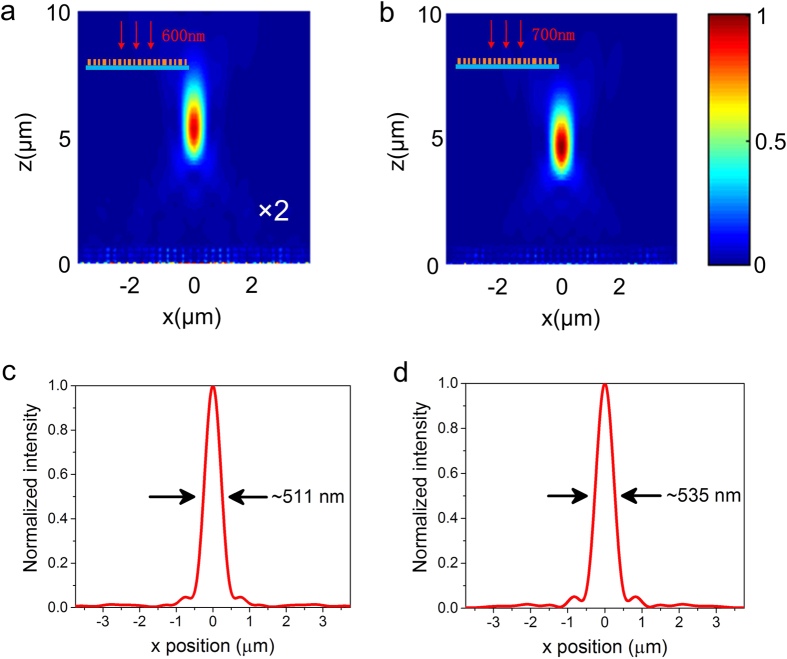
Focusing performance of the achromatic flat lens at the wavelength of (**a**) 600 nm and (**b**) 700 nm. The focal length is *f* = 5 μm. The field intensity shown in (**a**) is multiplied by 2 to obtain a similar contrast as (**b**). (**c**) and (**d**) Corresponding normalized intensity cross-sections at the flat lens’s focal spots along the *x* axis at the wavelength of 600 nm (**c**) and 700 nm (**d**). FWHMs of the focal spots are labelled on the plots.

As is well known, the conventional optical lens always suffers from chromatic aberrations due to the material’s dispersion. Here, a metasurface lens with superimposed phase profiles is employed to achieve achromatic focusing at two different wavelengths. The phase shift φ imposed on each point of the reflector-arrays depends on the composition of the phase profiles of two different wavelengths. Therefore, the phase function can be given as:


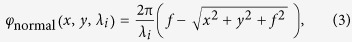


where *λ*_*i*_ represents different incident wavelength. Based on Eq. 3, an achromatic flat lens with a focal length of 5 μm and working at the dual-wavelengths (*λ*_1_ = 600 nm, *λ*_2_ = 700 nm) is designed (see Supplementary Fig. S3 for the phase distribution and corresponding structure of the flat lens). The achromatism of the lens is numerically demonstrated in Fig. 6(a)–(d). The normal incident RCP light at wavelength of 600 nm and 700 nm both get well focusing near the designed position. The difference of the energy between two focal points is mainly attributed to the different polarization conversion efficiencies for two wavelengths. Although the design based on superimposed geometric phase will lead to reducing sampling and the efficiencies of multifocal and achromatic focusing, it is still an effective method to independently control the wavefront of light with different polarizations and wavelengths.

In conclusion, we design a visible light reflective focusing flat lens that is composed by the TiO_2_ nanofins associated with Ag mirror substrate. The hybrid dielectric-metal metasurface geometry makes the lens have excellent focusing performance with the spot size close to the diffraction limit. The reflective flat lens is able to focus visible light at arbitrary position above the metasurface via tuning phase profile generated by the nanofin reflector-arrays. In addition, various functionalities including multifocal and achromatic focusing are achieved for the flat lens based on superimposed geometric phase design. We envision that this type of metasurface flat lenses will find the potential applications in the visible light imaging and sensing systems.

## Methods

### Simulation of polarization conversion efficiency

The 3D finite-difference time-domain (FDTD) simulations are performed for the designed unit cell with an area of 250 × 250 nm^2^ in *x-y* plane using periodic boundary conditions. Perfectly matched layers (PML) conditions are employed along the propagation of incident light (*z*-axis). The mesh size added on our nanostructure is *d*_*x*_ = *d*_*y*_ = *d*_*z*_ = 5 nm, which can ensure the accurate results. A linearly polarized plane wave is normally incident to the unit cell along the *z* direction. The reflection coefficients for circularly polarized light are calculated as 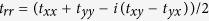 and 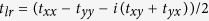
18, in which *t*_*xx*_, *t*_*yy*_, *t*_*xy*_, *t*_*yx*_ are reflection coefficients for linearly polarized light. The refractive index of TiO_2_ is utilized from the data of reference^22^ (~2.4).

### Simulation of the metasurface flat lens

By employing PML conditions, the designed flat lens with a total area of 7.75 × 7.75 μm^2^ is simulated in a space of 8.75 × 8.75 × 10 μm^3^ simulation region. All the proposed flat lenses have the same size. To obtain circularly polarized light, two orthogonal linearly polarized light sources with phase difference of 90 degree are added at the same position in the FDTD simulation region. The mesh size added on the whole flat lens is *d*_*x*_ = *d*_*y*_ = *d*_*z*_ = 5 nm. Electric intensity distributions are recorded in *x-y* and *x-z* plane at the designed focal position.

## Additional Information

**How to cite this article:** Fan, Q. *et al*. Visible light focusing flat lenses based on hybrid dielectric-metal metasurface reflector-arrays. *Sci. Rep.* **7**, 45044; doi: 10.1038/srep45044 (2017).

**Publisher's note:** Springer Nature remains neutral with regard to jurisdictional claims in published maps and institutional affiliations.

## Supplementary Material

Supplementary Information
