# Supplementary material for: Visible light focusing flat lenses based on hybrid dielectric-metal metasurface reflector-arrays
Source: Sci Rep. 2017 Mar 23;7:45044. doi: 10.1038/srep45044 (PMC5362929; doi:10.1038/srep45044)
Supplement: Supplementary Information [file srep45044-s1.pdf]

Visible light focusing flat lenses based on hybrid dielectric-metal metasurface reflector-arrays
Supplementary information

Qingbin Fan[1], Pengcheng Huo[1], Daopeng Wang[1], Yuzhang Liang[1], Feng Yan[2] and Ting Xu[1]*

1. National Laboratory of Solid State Microstructures, College of Engineering and Applied Sciences and Collaborative Innovation Center of Advanced Microstructures,
2. School of Electronic Science and Engineering, Nanjing University
22 Hankou Road, Nanjing 210093, China.
**xuting@nju.edu.cn*

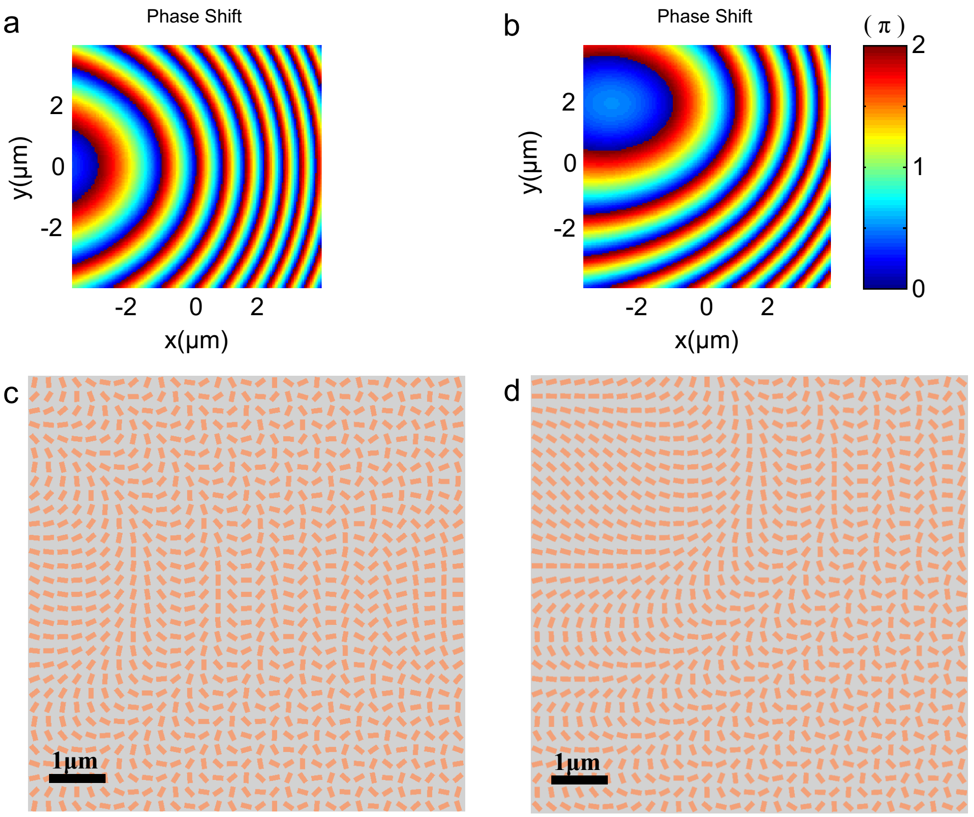

Figure S1. The ideal phase shift distribution of the designed lens to focus at (a) central position (x=0, y=0) and (b) arbitrary position (x=2 μm, y=2 μm) in the x-y plane. (c and d) Corresponding structure of the flat lens.

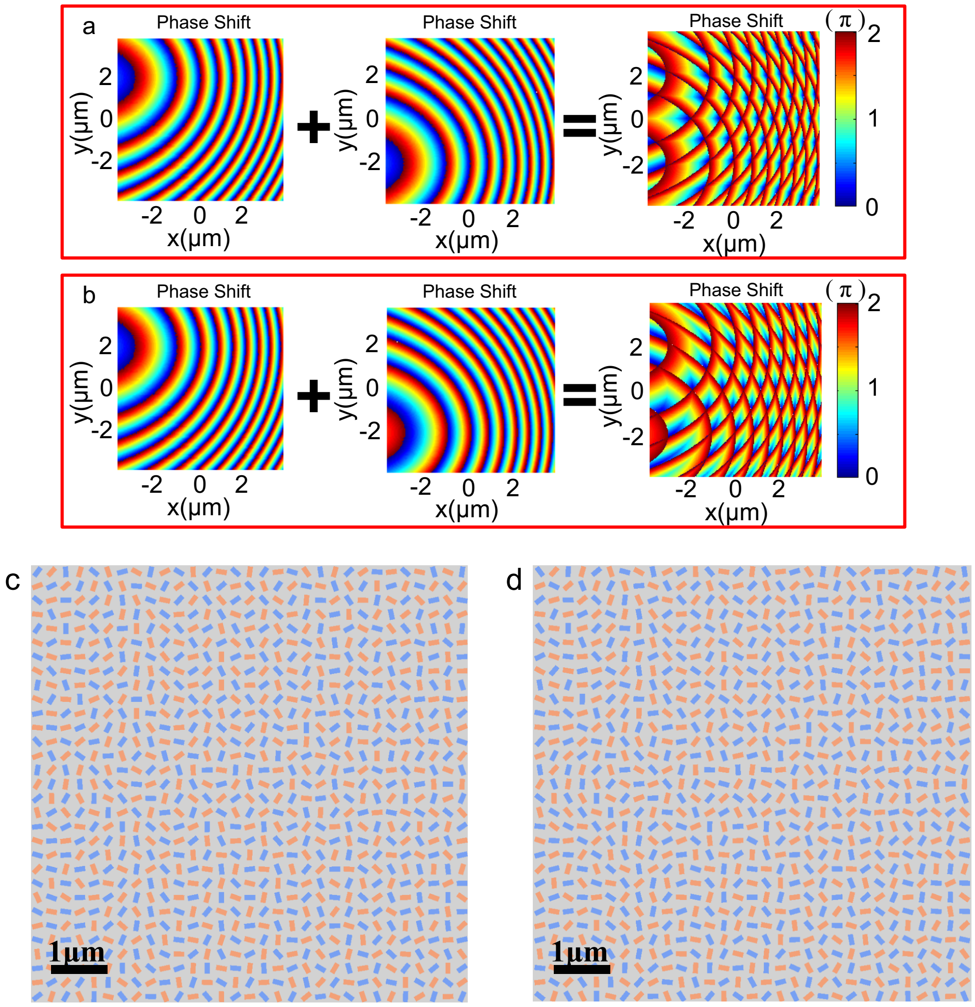

Figure S2. Illustration of the formation principle of the composited lens's phase shift distribution for multifocal focusing with the (a) same and (b) opposite chirality. (c and d) Corresponding structure of the flat lens.

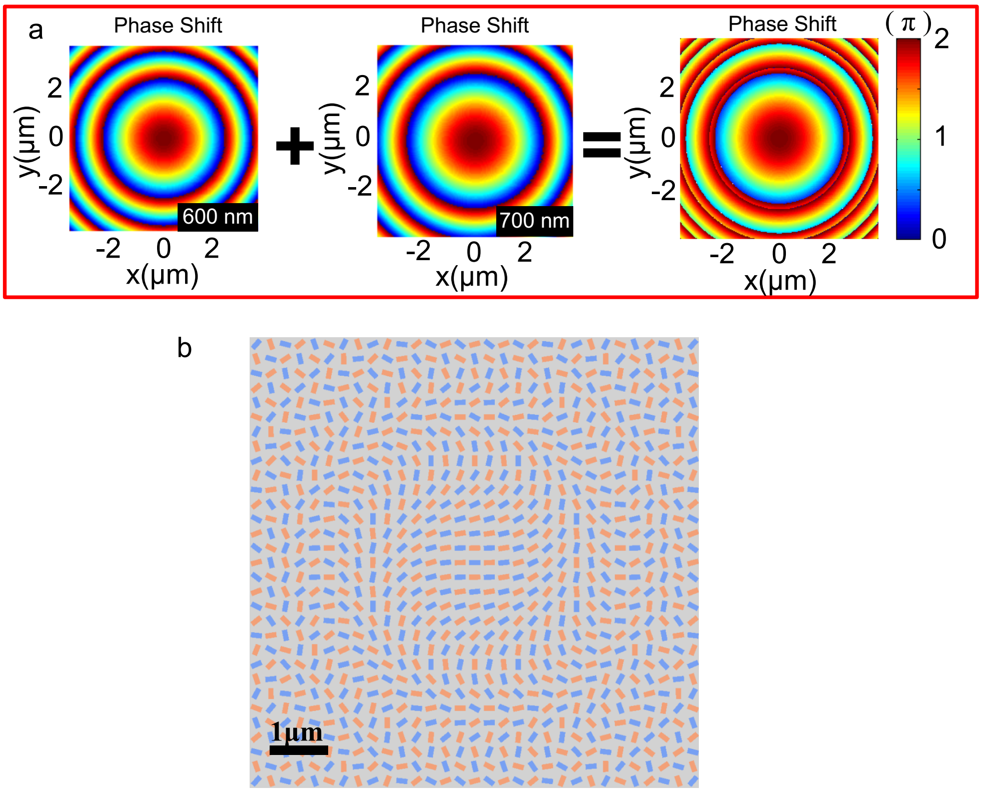

Figure S3. (a) Illustration of the formation principle of the achromatic lens's phase shift distribution. (b) Corresponding structure of the flat lens.
